# Citation of updated and co-published Cochrane Methodology Reviews

**DOI:** 10.1186/s13643-023-02270-w

**Published:** 2023-07-14

**Authors:** Linlin Zhu, Ziyu Yang, Hongyu Deng, Yonggang Zhang, Xiaoyang Liao, Mike Clarke

**Affiliations:** 1 General Practice Ward/International Medical Center Ward, General Practice Medical Center and National Clinical Research Center for Geriatrics, West China Hospital, Sichuan University, Chengdu, 610041 China; 2 Department of Periodical Press and National Clinical Research Center for Geriatrics, West China Hospital, Sichuan University, Chengdu, 610041 China; 3 Northern Ireland Methodology Hub, Centre for Public Health, Queen’s University Belfast, Belfast, BT12 6BJ Northern Ireland

**Keywords:** Cochrane reviews, Methodology, Update, Citations, Co-publication, Journal impact factor

## Abstract

**Background:**

To evaluate the number of citations for Cochrane Methodology Reviews after they have been updated or co-published in another journal, and the effect of co-publishing the review on the co-publishing journal’s impact factor (IF).

**Methods:**

We identified all Cochrane Methodology Reviews published in the *Cochrane Database of Systematic Reviews* (*CDSR*) before 2018 and searched for co-published versions in the Web of Science Core Collection database up to 16 August 2022. The included reviews were in two cohorts: those that had been published and updated in *CDSR* and those that had been published in *CDSR* and co-published in another journal. The primary outcome measured the citation number to updated and original reviews in the first five years after publication of the updated review, and assessed the citation number of co-published and non-co-published reviews in the first five years after publication of the co-published version.

The secondary outcome was the ratio of an adjusted IF and the actual IF of the co-publishing journal.

**Results:**

Eight updated and six original reviews were identified for the updated cohort of reviews, and four co-published reviews were included in the co-published cohort. The original reviews continued to be cited after the update was published but the median for the total number of citations was non-significantly higher for the updated reviews than for their original version[161 (Interquartile range (IQR) 85, 198) versus 113 (IQR 15, 433)]. The median number of total citations [362 (IQR 179, 840) versus 145 (IQR 75, 445)] and the median number of citations to the review in the first five years after co-publication combined and in each of those years was higher in the co-published group than in the non-co-published group. One of the three journals that co-published Reviews in the first year and two journals in the second year had a lower IF after co-publication.

**Conclusions:**

Earlier versions of Cochrane Methodology Reviews continue to be cited after an update is published, which raises doubts about whether those citing are using the most recent evidence or are aware of the update. Co-publication facilitates broader application and dissemination of Cochrane methodology evidence.

## Background

Systematic reviews can facilitate decision-making by relevant professionals, policy makers and the public and they need to be kept up to date if new studies would change their conclusions. Reviews that are out of date might provide misleading information [1] due to the absence of the latest evidence, and cause cascading damage to the quality of research [2]. Since its inception, the Cochrane Collaboration (now, Cochrane) has been dedicated to the periodic updating of Cochrane Reviews [1] with an update defined as “a new edition of a published Cochrane review with changes that can include new data, new methods, or new analyses to the previous edition” [3]. These updates include an updated search for eligible studies [4] and are marked to indicate whether or not the updating led to a change in the review’s conclusions [4]. An update of a Cochrane Review can provide users with the latest outcomes or conclusions from the evidence and, even without any changes in the results or conclusions, the update can reassure users that no recent evidence is missing [1]. However, the original Cochrane Review might continue to be cited and used after the update is published. Bodil et al. found that twenty-five percent of Cochrane reviews were still cited 10 years after last update and were on average cited 4.3 times in the 10^th^ year [5].

Cochrane allows co-publication of Cochrane Reviews in other journals in certain circumstances [6]. Co-publication is not a duplicate publication or academic misconduct, but rather the co-published version is a secondary publication of the Cochrane Review [7], and should be peer reviewed and edited according to the co-publishing journal’s editorial process. Co-publication of Cochrane Reviews should be carried out with the agreement of Cochrane and the co-publishing journals [8], and might take the form of be an abridged version of the full review, a “Cochrane corner” of a journal (where the summary of the review is accompanied by commentary), or a short version translated into a language other than English [6].

In an earlier study, the annual co-publication rate for Cochrane Reviews fluctuated from 0.96% to 3.94% [9] between 2005 and 2015. It has also been shown to vary across different Cochrane groups. For example, 19.6% of reviews from the Cochrane Eyes and Vision Group were co-published in 2007, and 16.2% in 2014 [10, 11]. Co-publication of Cochrane Reviews in other journals might promote access to the evidence and increase citations [10, 12] and journals that co-publish Cochrane Reviews might also increase their impact factor (IF) [12]. Our previous study [9] found that the total number of citations for co-published Cochrane Reviews (combining the citations for the original Cochrane Review and the co-published version) was significantly higher than that for non-co-published reviews. This showed that co-publication not only improved the dissemination and accessibility of Cochrane evidence but also facilitated its uptake.

In the study reported here, we investigated the effect of updating and of co-publication on the number of citations to Cochrane Methodology Reviews. Most Cochrane Reviews relate to the effects of health and social care, but Cochrane Methodology Reviews are a subset focused on research into the methods used in research into health and social care. We have compared citations to the updated and the original Cochrane Methodology Review in the years after the update was published, the citations for co-published and non-co-published Cochrane Methodology Reviews and the effect of co-publishing the review on the co-publishing journal’s IF.

## Methods

### Study design

This was a retrospective study of Cochrane Methodology Reviews. In August 2022, we identified all Cochrane Methodology Reviews in the *Cochrane Database of Systematic Reviews* (*CDSR*) in the Cochrane Library (https://www.cochranelibrary.com/) which had been published before 31 December 2018. We searched for co-published versions of these reviews in another journal in the Web of Science Core Collection database up to 16 August 2022. The number of citations for each version of the Cochrane Methodology Review and any co-published versions was also obtained from the Web of Science Core Collection database on 16 August 2022.

The included reviews were divided into two cohorts: (1) those that had been updated before 2018 and (2) those that had been co-published in another journal. For the updated cohort, the most recent versions or previous version of the updated Cochrane Methodology Reviews were the updated group and the original review were original group. If a review is updated multiple times, each updated version is included in the updated group. For the co-published cohort, reviews which were co-published in another journal at the same time or later than the original review were the co-publication group, and the Cochrane Methodology Reviews had not been co-published were non-co-publication group.

### Inclusion and exclusion criteria

Inclusion criteria were (1) Cochrane Methodology Reviews that had been updated before 2018; or (2) Cochrane Methodology Reviews that had been published before 2018 and co-published in another journal at the same time or after the publication of the Cochrane Methodology Review in the *CDSR*. Exclusion criteria were (1) Cochrane Methodology Reviews that had not been updated or co-published before 2018; and (2) Cochrane Reviews or updated versions or co-published versions for which the number of citations could not obtained. Although there are examples of co-publication of Cochrane Methodology Reviews after 2018 [13, 14], we restricted this study to reviews published before 2018 in order to be able to investigate the trajectory of citations in the five years after updating or co-publication.

### Review selection and data extraction

Two authors (LLZ and ZYY) reviewed and extracted data independently. Disagreements were resolved by consensus, with arbitration by a third author (HYD) if necessary. A standardized extraction form was used to collect the following data for eligible reviews: title, number of included studies, results and conclusions, authorship of *CDSR* and co-published versions, journal of co-publication, time interval between original and updated or co-published review. The number of citations to the Cochrane Methodology Reviews and to the co-published versions, and the IF of the co-publishing journal were taken from the Web of Science database (Journal Citation Report).

### Outcomes

The primary outcomes were the average (mean and median) number of citations to the updated and the original Cochrane Methodology Reviews in the five years after the update had been published and the average number of citations to the co-published and non-co-published Cochrane Methodology Reviews in the first five years after co-publication. The secondary outcome was the ratio of an adjusted IF (excluding the data for the co-published Cochrane Methodology Review) and the actual IF of the co-publishing journal.

We calculated the number of citations for each co-published review as the sum of the citations to the Cochrane Methodology Review and to its co-published version. The journal’s actual IF is calculated by:

$$\mathrm{IF}=\frac{\mathrm{Citations\ in\ year\ {\rm X}}\mathrm{\ to\ items\ published\ in\ year\ }\left(\mathrm{\rm X}-2\right)\ +\ \mathrm{Citations\ in\ year\ {\rm X}}\mathrm{\ to\ items\ published\ in\ year\ }\left(\mathrm{\rm X}-1\right)}{\mathrm{Number\ of\ citable\ items\ in\ year\ }\left(\mathrm{\rm X}-2\right)\ +\ \mathrm{Number\ of\ citable\ items\ in\ year}\ \left(\mathrm{\rm X}-1\right)}$$

Our adjusted IF was calculated by:

$$\mathrm{Adjusted\ IF}=\frac{\mathrm{Citations\ in\ year\ {\rm X}}\mathrm{\ to\ items\ published\ in\ year }\left(\mathrm{\rm X}-2\right)\ +\ \mathrm{A }}{\mathrm{Number\ of\ citable\ items\ in\ year\ }\left(\mathrm{\rm X}-2\right)\ +\ \mathrm{B}}$$

$$\mathrm{\rm A}=\left[\mathrm{Citations\ in\ year\ {\rm X}}\mathrm{\ to\ items\ published\ in\ year\ }\left(\mathrm{\rm X}-1\right)\right]-[\mathrm{Citations\ in\ year\ {\rm X}}\mathrm{\ to\ Cochrane\ review\ copublications\ in\ year\ }\left(\mathrm{\rm X}-1\right)]$$

$$\mathrm{\rm B}=\left[\mathrm{Number\ of\ citable\ items\ in\ year}\left(\mathrm{\rm X}-1\right)\right]-\mathrm{\ Number\ of\ Cochrane\ review\ copublications}$$

We then calculated a ratio by dividing the adjusted IF by the journal’s actual IF. If this ratio was greater than or equal to 1, the co-published reviews did not increase the journal IF; while values less than 1 indicated that the co-published reviews increased the journal IF. When more than one co-publication of a Cochrane Methodology Review was identified in a single journal, we calculated the mean ratio of IF and its 95% confidence interval (CI).

### Statistical snalysis

We used the Fisher’s exact test to compare categorical variables, the Student t-test for continuous and normally distributed variables and the Mann–Whitney U-test to compare medians if the data were not normally distributed. We conducted a normality test on the data using a histogram and a normal P-P plot. A *p*-value of < 0.05 was considered statistically significant. We used SPSS 26.0 software for the statistical analyses.

## Results

### Baseline characteristics of Cochrane reviews

**Fig. 1:**
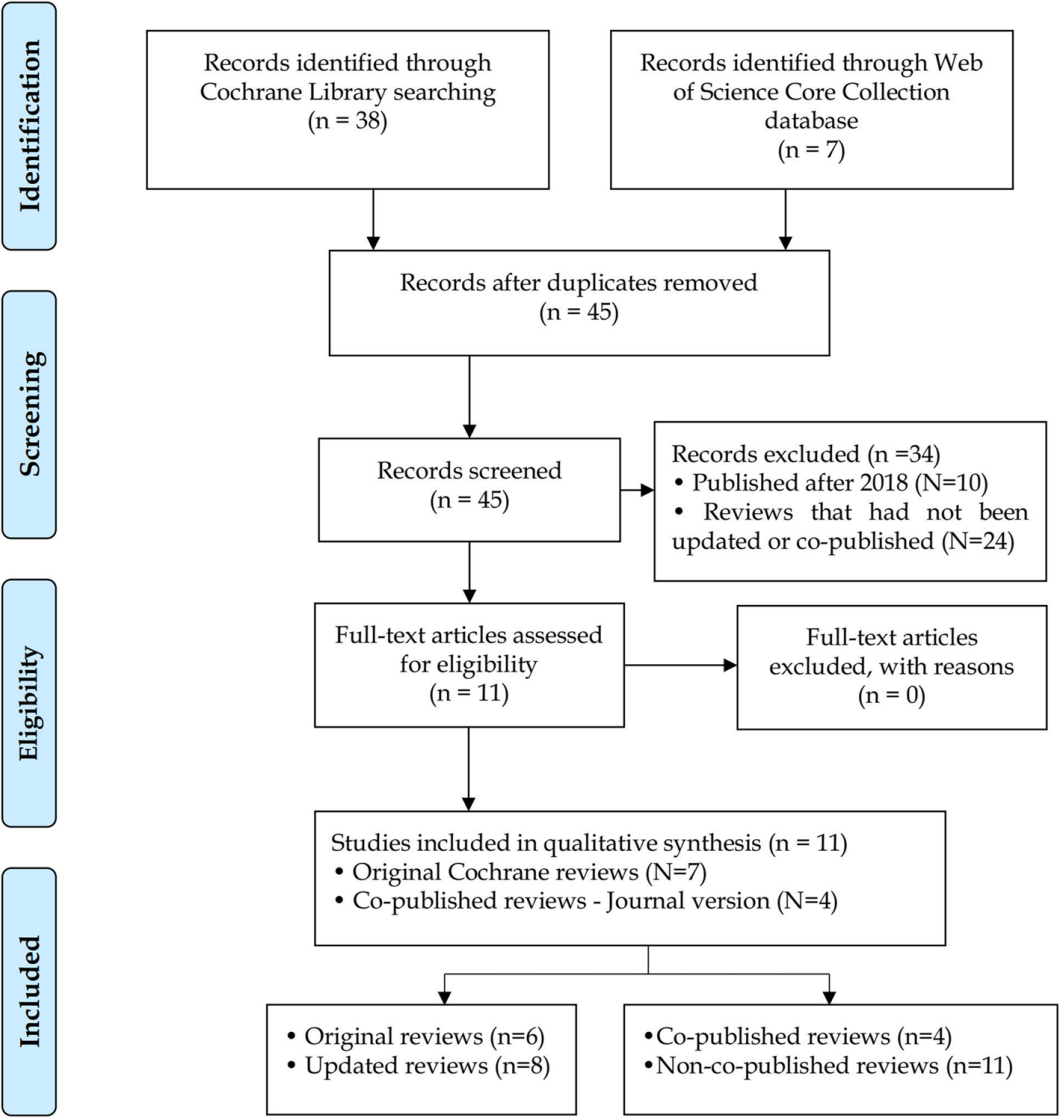
The flow diagram of included Cochrane Methodology Reviews

There were 38 full Cochrane Methodology Reviews in *CDSR* on 16 August 2022, which had been updated between zero and five times by that date. Six of these reviews [15–20] had been updated [21–26] before 2018. We also retrieved four versions of Cochrane Methodology Reviews (two original reviews [17, 27] and two updated reviews [23, 28]) that had been co-published in another journal [29–32] before 2018 from the Web of Science Core Collection database on 16 August 2022. In total, our sample comprises 14 published versions of Cochrane Methodology Reviews and four co-published articles. The flow diagram is shown in Fig. 1.

We categorized the included records into two cohorts as described above. In the updated cohort, the updated group included 8 updated reviews, while the original group included 6 original Cochrane Methodology Reviews. The co-published cohort consisted of 4 co-published and 11 non-co-published Cochrane Methodology Reviews. The included reviews had been published between 2007 and 2018. The main characteristics of the two cohorts are shown in Tables 1 and 2.

**Table 1:** Characteristics of the updated and original Cochrane Methodology Reviews

Study characteristics	Updated *N* = 8	Original *N* = 6	*P* value
Year of publication			
2007 to 2010	4	5	0.301
2011 to 2018	4	1	
Number of authors			
Mean ± SD	8.0 ± 3.4	4.2 ± 2.6	0.039
Number of included studies			
Mean ± SD	146.4 ± 190.8	94.0 ± 138.2	0.581
Median (IQR)	56.5 (28.3, 337.5)	40 (17.3, 152.3)	0.518

**Table 2:** Characteristics of the co-published and non-co-published Cochrane Methodology Reviews

Study characteristics	Co-published *N* = 4	Non-co-published *N* = 11	*P* value
Year of publication in CDSR			
2007 to 2010	1	8	0.235
2011 to 2018	3	3	
Number of authors			
Mean ± SD	7.3 ± 3.3	6.1 ± 3.6	0.566
Number of included studies			
Mean ± SD	51.5 ± 16.2	142.5 ± 184.8	0.136
Median (IQR)	46.5 (39.8, 68.3)	32.0 (18.0, 372.0)	0.695

### Characteristics of updated and co-published Cochrane review

Topics addressed in the eight updated Cochrane Methodology Reviews included issues relating to randomization and recruitment to randomized trials (4 reviews) [25, 26, 28, 33], conflict of interest (1 review) [23], technical editing (1 review) [21], publication (1 review) [22] and questionnaire response (1 review) [24]. Five (62.5%) had been updated only once before 2018 [21–25], while the other had been updated three times [26, 28, 33]. The interval time between versions of the reviews varied from 0.5 year to 11 years. Characteristics are summarized in Table 3.

**Table 3:** Characteristics of eight updated Cochrane Methodology Reviews

Updated review characteristics	N (%)
Authorship of updated review	
Identical to original review [21, 24]	2 (25.0%)
Different authors and different order [26, 28, 33]	3 (37.5%)
Authors added or removed [22, 23, 25]	3 (37.5%)
Number of updates	
One [21–25]	5 (62.5%)
Three [26, 28, 33]	3 (37.5%)
Interval between original and updated review	
One year [21]	1 (12.5%)
Two years [24]	1 (12.5%)
Three years [28, 33]	2 (25.0%)
Four years [25]	1 (12.5%)
Five years [23]	1 (12.5%)
Eleven years [22, 26]	2 (25.0%)
Methodological topic of included studies	
Randomization method [25, 26, 28, 33]	4 (50.0%)
Conflict of interest [23]	1 (12.5%)
Technical editing [21]	1 (12.5%)
Full publication of results [22]	1 (12.5%)
Questionnaires [24]	1 (12.5%)
Conclusions	
Same as previous version [21–25]	5 (62.5%)
Different to previous version [26, 28, 33]	3 (37.5%)

The four co-published Cochrane Methodology Reviews were co-published in three journals. Three (75%) were co-published more than two years after the publication of the Cochrane Methodology Review. The types of co-publication were mostly a short version of the full review (3 reviews), with one being a specially prepared summary of the review. One Cochrane Methodology Review had a co-publication for both its original and updated version. The other two co-publications were for the original version of the Cochrane Methodology Review (1 review) and for the updated review (1 review). For all four cases, the results and conclusions were the same between the Cochrane Methodology Review and the co-published version. Characteristics are summarized in Table 4.

**Table 4:** Characteristics of four co-publications (journal version)

Co-publications characteristics	N (%)
Authorship of co-publication	
Identical to Cochrane Methodology Review [32]	1 (25.0%)
Same authors, different order [29, 30]	2 (50.0%)
Authors added or removed [31]	1 (25.0%)
Co-publication timing	
Year after publication of Cochrane Methodology Review [29, 31, 32]	3 (75.0%)
> 2 years after publication of Cochrane Methodology Review [30]	1 (25.0%)
Co-publication content compared to full Cochrane Methodology Review	
Short version^a^ [29, 30, 32]	3 (75.0%)
Review summary^b^ [31]	1 (25.0%)
Number of included studies	
Same as Cochrane review [29, 30, 32]	3 (75.0%)
Less than Cochrane review [31]	1 (25.0%)
Conclusions	
Same as Cochrane review [29–32]	4 (100.0%)

^a^Short version of a Cochrane Review: this includes republishing a part of the review (such as the abstract, plain language summary) or an abridged version [34]

^b^Review summaries are summaries of a Cochrane review where the authors of the article provide a commentary on the Cochrane review in their own words

### Number of citations for updated and original Cochrane Methodology Reviews

As shown in Table 5, the original Cochrane Methodology Review was still being cited after the updated Cochrane review had been published in all six cases and, in one, there were 485 citations to the original version of the review and only 105 to the update in the five years after publication of the update. Combining all the reviews and considering the total number of citations between the publication date of the updated version and 16 August 2022, this was non-significantly higher for the updated Cochrane Reviews than for their original version [161 (IQR 85, 198) versus 113 (IQR 15, 433), *p* = 0.518]. There were also non-significantly more citations to the updated review than the original review if this analysis is limited to the first five years after publication of the update [78 (IQR 61, 149) versus 91 (IQR 14, 247), *p* = 0.897]. Table 6 shows that in the first three years after the publication of the update, the median number of citations to the updated review was lower than to the original review but it was higher in each of the next two years.

**Table 5:** Total number of 5-year citations for each of the updated reviews

*Total number of citations in the first 5 years after update*	Original review	Updated review	*p*-value
Wager 2007 [15, 21]	2	12	0.474
Scherer 2007 [16, 22]	126	75	
Lundh 2012 [17, 23]	485	105	
Edwards 2007 [18, 24]	168	164	
Kunz 2007 [19, 25]	55	56	
Mapstone 2007 [20, 26, 28, 33]	18	78	
		78	
		177	
Mean ± SD	93.1 ± 54.7	145.5 ± 177.0	
Median (IQR)	78 (61, 149)	91 (14, 247)	0.897

**Table 6:** Number of citations for the updated and original Cochrane reviews after the updated year

Average number of citations	Updated *N* = 8	Original *N* = 6	*p*-value
*Total number of citations between update and 16 August 2022*			
Mean ± SD	197.4 ± 188.4	202.3 ± 225.5	0.965
Median (IQR)	161 (85, 198)	113 (15, 433)	0.518
*Total number of citations of the first five years after update*			
Mean ± SD	93.1 ± 54.7	145.5 ± 177.0	0.474
Median (IQR)	78 (61, 149)	91 (14, 247)	0.897
*Year of publication of update*			
Mean ± SD	4.6 ± 8.3	31.2 ± 37.9	0.076
Median (IQR)	2 (0, 4)	20 (8, 51)	0.069
*Second year after update*			
Mean ± SD	14.8 ± 9.6	29.2 ± 29.6	0.295
Median (IQR)	14 (7, 21)	24 (6, 50)	0.560
*Third year after update*			
Mean ± SD	20.0 ± 14.4	33.5 ± 39.5	0.386
Median (IQR)	18 (9, 35)	19 (6, 63)	0.796
*Fourth year after update*			
Mean ± SD	28.8 ± 23.8	30.3 ± 46.2	0.934
Median (IQR)	21 (10, 49)	13 (4, 53)	0.366
*Fifth year after update*			
Mean ± SD	25.0 ± 15.0	21.3 ± 26.2	0.745
Median (IQR)	30 (10, 34)	10 (5, 42)	0.364

### Number of citations for co-published and non-co-published Cochrane Methodology Reviews

The median for the total number of citations (combining citations to the original Cochrane Methodology Review and to its co-publication) up to 16 August 2022 was non-significantly higher in the group of co-published reviews than in the non-co-published group [362 (IQR 179, 840) versus 145 (IQR 75, 445), *p* = 0.090]. Similarly, the median number of citations in each of the five years (Table 7) and sum of the first five years after publication in the co-published group was higher than in the non-co-published group [177 (IQR 99, 338) versus 75 (IQR 37, 126); *p* = 0.037].

**Table 7:** Number of citations for the co-published and non-co-published reviews

Average number of citations	Co-published *N* = 4	Non-co-published *N* = 11	*p*-value
*Total number of citations to 16 August 2022*			
Mean ± SD	460.3 ± 360.2	220.6 ± 207.3	0.126
Median (IQR)	362 (179, 840)	145 (75, 445)	0.090
*Total number of citations in the first five years after co-publication*			
Mean ± SD	204.5 ± 129.8	82.5 ± 58.4	0.023
Median (IQR)	177 (99, 338)	75 (37, 126)	0.037
*Year of publication*			
Mean ± SD	9.8 ± 12.8	2.1 ± 2.7	0.317
Median (IQR)	6 (1, 23)	1 (0, 3)	0.286
*Second year after publication*			
Mean ± SD	36.0 ± 26.6	12.8 ± 10.3	0.025
Median (IQR)	29 (16, 63)	13 (5, 17)	0.067
*Third year after publication*			
Mean ± SD	49.3 ± 40.0	19.7 ± 14.3	0.046
Median (IQR)	36 (22, 90)	19 (9, 34)	0.117
*Fourth year after publication*			
Mean ± SD	47.0 ± 33.0	26.5 ± 21.5	0.178
Median (IQR)	37 (23, 82)	22 (10, 34)	0.170
*Fifth year after publication*			
Mean ± SD	62.5 ± 36.2	21.4 ± 16.9	0.104
Median (IQR)	59 (30, 99)	15 (8, 32)	0.050

*IQR* Inter-quartile range

### Number of citations for the Cochrane Methodology Review and its co-publication

As shown in Table 8, in the five years after co-publication, the original Cochrane Methodology Review received more citations than the co-published version for two reviews while the co-published version received more citations for the other two reviews. The median for the number of citations was non-significantly higher for the original Cochrane Methodology Review than for its co-published version for the total number of citations up to 16 August 2022 [157 (IQR 102, 720) vs 85 (IQR 54, 265), *p* = 0.248], for the number of citations in the first five years after co-publication [92 (IQR 45, 290) versus 49 (IQR 37, 102)] and for each of those five years (Table 9).

**Table 8:** Total number of 5-year citations for each of the co-published reviews

*Total number of citations in the first five years*	Cochrane Review	Co-published version	*p*-value
Brueton 2013 [27, 29]	34	45	0.322
Treweek 2010 [28, 30]	78	118	
Lundh 2012 [17, 31]	351	34	
Lundh 2017 [23, 32]	105	53	
Mean ± SD	142.0 ± 142.4	37.8 ± 18.9	
Median (IQR)	92 (45, 290)	49 (37, 102)	0.468

**Table 9:** Number of citations for the Cochrane Methodology Review and its co-publication

Average number of citations	Cochrane review *N* = 4	Co-published version *N* = 4	*p*-value
*Total number of citations up to 16 August 2022*			
Mean ± SD	326.0 ± 381.2	134.3 ± 123.8	0.322
Median (IQR)	157 (102, 720)	85 (54, 265)	0.248
*Total number of citations in the first five years*			
Mean ± SD	142.0 ± 142.4	62.5 ± 37.8	0.376
Median (IQR)	92 (45, 290)	49 (37, 102)	0.468
*Year of co-publication*			
Mean ± SD	7.0 ± 12.0	2.8 ± 3.1	0.519
Median (IQR)	2 (0, 19)	2 (0, 6)	1.000
*Second year after co-publication*			
Mean ± SD	28.0 ± 29.5	8.0 ± 5.0	0.230
Median (IQR)	18 (7, 59)	7 (4, 13)	0.245
*Third year after co-publication*			
Mean ± SD	32.0 ± 42.2	17.3 ± 7.5	0.517
Median (IQR)	13 (8, 75)	16 (11, 25)	0.564
*Fourth year after co-publication*			
Mean ± SD	31.3 ± 37.6	15.8 ± 9.1	0.454
Median (IQR)	15 (8, 71)	15 (8, 25)	0.773
*Fifth year after co-publication*			
Mean ± SD	43.8 ± 36.9	18.8 ± 14.9	0.255
Median (IQR)	33 (17, 81)	12.0 (10, 34)	0.248

### Effect of co-publication on journal impact factor

The four co-published Cochrane Methodology Reviews were co-published in three journals (Table 10). Among these three journals, the ratio of the adjusted IF to the actual IF was less than 1 in one journal for the first year after publication, and less than 1 in two of the three journals in the second year after publication. This means that the co-published review increased the IF for these journals in those years.

**Table 10:** Ratio of the adjusted IF to the actual IF

Journal name	Ratio—first year after Co-published Mean ± SD (95% CI)	Ratio—second year after Co-published Mean ± SD (95% CI)
Intensive Care Med^a^	1.003	0.999
BMJ Open	0.998 ± 0.002(0.981, 1.015)	0.997 ± 0.002 (0.975, 1.019)
JAMA Imtern Med^a^	1.005	1.000

^a^There was only one result (IF ratio) for these two journals so we could not calculate a SD and 95% CI

## Discussion

This study is the first that we are aware of that investigates the citing of updated and original Cochrane Methodology Reviews after an update has been published, and the impact of co-publication for this type of Cochrane Review. Our results show that the original Cochrane Methodology Review was still being cited after its updated version had been published and, in some cases, there were more citations to the original review than to the update in the five years after the update was published.

We speculate that there are several reasons for the original version of a Cochrane Methodology Review to continue to be cited after an update is published. For instance, the citing authors may have a preference based on the conclusions of the two versions of the review [35], they may be more familiar with the original version or might not have been aware of the updated version at the time they prepared their article, they might be referring to something in the original version that was not included in the update or they might be re-using text that they have written previously. It is also possible that the cited Cochrane Methodology Review was updated between the submission and acceptance of the citing article [36]. Another possible explanation is “second-hand” citation of the Cochrane Methodology Review based on it being cited elsewhere before the update [36], with the citing author using the citation from another paper without checking the original [37]. Actually, this does not comply with the International Committee of Medical Journal standards [38].

It is also worth noting that the number of citations for the updated Cochrane Methodology Reviews were much higher than those for the original version in the fourth and fifth year after the update was published, which might suggest that there is a lag of a few years in the uptake of evidence from Cochrane Methodology Reviews into the wider literature. This is in accordance with academic productivity trends, which have found that there were more citations from 2.5 to 5 years after publication compared with the first 2.5 years [39].

Turning to co-publication, the number of citations for co-published Cochrane Methodology Reviews is higher than that for non-co-published reviews at least for the first five years after co-publication, with the number of citations to the co-published version being higher than the number of citations to the Cochrane Methodology Review in two cases and lower in two cases. This is consistent with a previous study [9, 10] and suggests benefits for dissemination of the findings of Cochrane Reviews. First, if the co-publishing journal has a specialist audience, the Cochrane evidence would be brought to that audience [40–42] and some previous studies have shown that meta-analyses have higher rates of citation in specialty journals [39, 43–45]. Second, the higher number of citations suggest that the evidence in the Cochrane Review will have received more attention because of the co-publication. Third, the co-published version might make the Cochrane evidence more accessible to potential users than the full Cochrane Review and might be more useful to them as a reference source [46]. Among the three journals that co-published a Cochrane Methodology Review, the IF was raised by this in the first year in one journal and in the second year in two journals. This is also consistent with a previous study [9, 10]. The longer term effects on journals are less clear because of how the IF is calculated but citation rate peaks have been shown to vary across different journals, ranging from 2.5 to 7.2 years after publication [47, 48], which suggests that journals may benefit from co-publication beyond the two years used to calculate their IF [49].

Our study has some limitations. First, our sample may be too small to allow sufficiently powered analyses and conclusions, the results might be influenced by chance [50], and the generalisability to other types of Cochrane Review needs to be investigated in further research. Second, we only included data on citations and co-publications from the Web of Science Core Collection database, which may underestimate the true number of citations and may have missed some co-publications. Third, although some studies have found that meta-analyses have higher rates of citation, which may make it more likely that co-publishing a version of a Cochrane Review may have more impact on a journal’s IF than co-publishing of any other type of article, other studies have argued that study design does not significantly influence citation rate [51, 52]. For example, there may also be important associations with number of authors [53–55] or their geographic location [56]. These are issues that should be investigated in further research.

In conclusion, we have shown that the citation of updated Cochrane Methodology Reviews lags a few years after their publication and confirmed that co-publication increases the number of citations for this type of Cochrane Review and may increase the IF of the co-publishing journal. However, this raises issues about whether people wishing to use the evidence from Cochrane Methodology Reviews are always using the most up to date and complete information. This highlights the importance that authors who cite any previous research should confirm the validity of the reference and the need for ways to allow people to more clearly see that a review has been updated.

## Data Availability

The datasets used and/or analysed during the current study are available from the corresponding author on reasonable request.

## Abbreviations

IQR: Interquartile Range
SD: Standard Deviation
IF: Impact Factor
CDSR: Cochrane Database of Systematic Review*s*
